# Transforming our future by unlocking the power of DNA: the representation of health-related genomic testing in UK news items

**DOI:** 10.1038/s41431-026-02140-8

**Published:** 2026-05-21

**Authors:** Rachel Horton, Kendra Gordillo, Anneke Lucassen, Susie Weller

**Affiliations:** 1 Clinical Ethics, Law and Society group, Centre for Human Genetics, University of Oxford, Oxford, UK; 2 Centre for Personalised Medicine, Centre for Human Genetics, University of Oxford, Oxford, UK; 3 Wessex Clinical Genetics Service, Princess Anne Hospital, Southampton, UK; 4 Vrije Universiteit Amsterdam, Amsterdam, NL Netherlands

**Keywords:** Social sciences, Ethics, Patient education, Medical ethics

## Abstract

Many people will have had no formal education on health-related genomic testing, so information from media sources may constitute a major influence in shaping ideas and expectations around genomic tests. We undertook a framing analysis of 186 UK news items discussing health-related genomic testing in the context of various recent UK-based initiatives: the 100,000 Genomes Project; Our Future Health; the Generation Study; the Deciphering Developmental Disorders Study; and the NHS Genomic Medicine Service. We found that news items tended to frame genomic tests as the fruit of amazing technological progress. Tests were frequently described as ‘reading’ the genetic code and in the context of newborn screening, diagnosis and prediction were often treated as almost synonymous. Genomics was positioned as standing to give us all a healthier future. A decision to contribute genomic data to a research database was typically framed as virtuous, though some items discussed privacy concerns in depth. A clear diagnosis was achieved in 88% of instances where diagnostic genomic testing of a specific symptomatic person was discussed, with little attention afforded to those who had experienced less impressive outcomes. In summary, UK news items tended to celebrate the very best of genomic testing, but in doing so, they potentially engender an expectation that dramatically beneficial results come as standard. Providers of genomic tests need to be mindful of the backdrop of promotional media discourse when patients are making decisions around testing and ensure that consent conversations help patients anticipate potential limitations and challenges as well as benefits.

**Figure:**
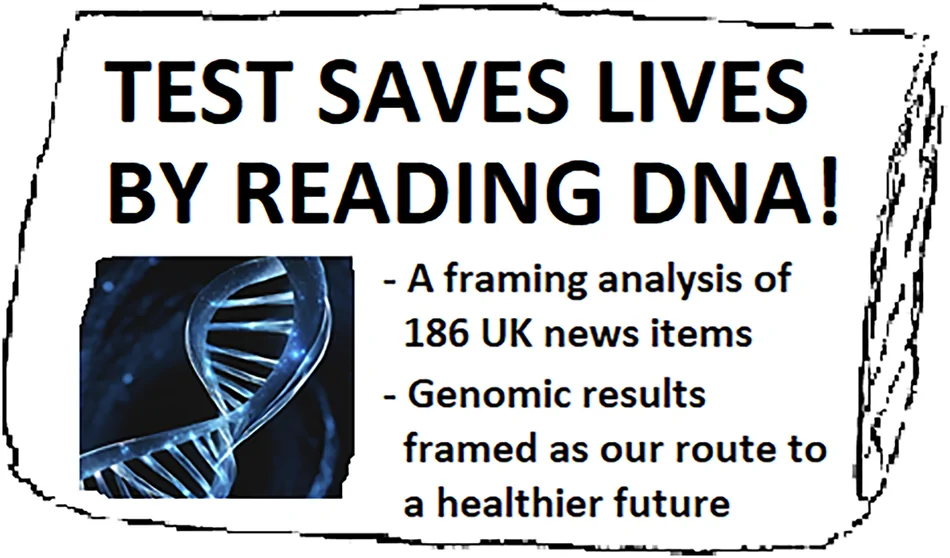

## Introduction

Genomic technology is playing an increasing role in healthcare. When used in diagnosing symptomatic rare disease, for the ~40% of people where it succeeds, this is often very useful. A diagnosis might allow more tailored clinical care, perhaps by informing prognosis or opening up new treatment options. It may have value in providing an explanation for a person’s health problems, or in clarifying reproductive options for their family [1]. Understandably, for some of the ~60% where diagnosis is not achieved, this may be experienced as a significant loss [2].

Recently, there has been considerable focus on the potential role genomics might play in the setting of population screening, for example, predicting rare disease in the newborn period [3], or susceptibility to a range of common diseases in adult life [4]. In a rare disease screening context, prior probabilities are very different from the investigation of existing, overt rare disease. Picking out which genomic variations might actually lead to disease is a very difficult task, as illustrated by studies delineating the range of seemingly abnormal variation in every ‘normal’ genome [5, 6]. In a common disease screening context, the small relative contribution of the genetic factors measured to overall risk means ‘high risk’ genetic results often have only slight impacts on absolute risk [7].

Yet despite its increasing importance and the subtleties associated with its interpretation in different contexts, many adults will not have had any formal education around the use of genomics in healthcare. Nevertheless, a recent international scoping review exploring the public’s knowledge of, attitude to and motivation towards health-related genomics indicated ‘*general positivity about genomics, with high awareness but little familiarity or factual knowledge. Publics had high expectations of genomics and perceived that it could provide them with information for their future*’ [8].

Where does this high awareness and general positivity come from? One potential source is media coverage of clinical genomics– Happer and Philo argue that ‘*The media play a central role in informing the public about what happens in the world, particularly in those areas in which audiences do not possess direct knowledge or experience*’ [9]. However, while most people accept that the media can influence us, there is much debate as to how and to what extent [10].

Condit’s work explores the complexity of people’s attitudes and shows that people strategically tap into different explanatory models depending on context. She reflects that ‘*Unqualified, direct sentences are quicker and easier to produce than qualified, multicausal sentences’* [11]. She illustrates that people might use a genetic essentialist explanatory model sometimes, yet in other contexts, draw on a different account, for example, highlighting the role of environmental factors [11].

This reminds us that the content of UK news items cannot be taken as a simple proxy for the attitudes of either writers or readers. However, we argue that the portrayal of clinical genomics in these news items still has the potential to influence people’s expectations and, as such, merits examination. The 2023 Ofcom report on news consumption in the UK noted that 96% of UK adults say they consume news in some form, with 68% consuming news via online sources [12]. So how have genomic tests and their results been framed in UK news?

## Methods

### Collecting news items

We used Google search^1^ to identify items from the BBC News and Sky News websites, together with five UK national newspaper titles. These sources were chosen based on their popularity [12] and aimed to encompass a range of political views and styles, outlined in Table 1 (newspaper selection influenced by Barkas et al. [13]).

**Table 1:** Sources searched for news stories.

News source	URL used for search	Political orientation	Style
BBC News	Bbc.co.uk/news	Neutral	Broadcast/online
Sky News	News.sky.com	Neutral	Broadcast/online
The Sun	Thesun.co.uk	Right of centre	Tabloid/online
The Guardian and Observer^a^	Theguardian.com	Left of centre	Broadsheet/online
Daily Mail and Mail on Sunday	Dailymail.co.uk	Right of centre	Midmarket/online
The Times and The Sunday Times	Thetimes.com	Right of centre	Broadsheet/online
The Mirror and The Sunday Mirror	Mirror.co.uk	Left of centre	Tabloid/online

^a^At the point the searches discussed in this article were undertaken, the Observer was owned by Guardian News and Media Ltd and operated as a sister paper to the Guardian. Ownership transferred to Tortoise Media in April 2025 (https://www.theguardian.com/info/2025/apr/07/observer-faqs).

We developed search terms aiming to elicit news items that discussed genomic testing in healthcare contexts. Initial broad searches yielded hundreds of results, most of which were not relevant to genomic testing to inform clinical care. In reading through some of the items identified, we found that those around clinical care tended to discuss or at least mention recent or current clinical genomic initiatives. We therefore developed searches aiming to elicit news items mentioning UK initiatives offering tests examining large amounts of genetic code. The initiatives and corresponding search terms are detailed in Table 2.

**Table 2:** UK initiatives included and terms used to collect relevant news items.

Initiative	Description	Search terms
100,000 Genomes Project	Launched in the UK in 2013 ‘*to apply whole-genome sequencing to the study of rare diseases, cancers and infections in a national health care setting*’ [8]. ~85,000 people joined the project between 2014 and 2018. The project had a ‘rare disease’ arm, aiming to find molecular diagnoses for people and families affected by rare genetic conditions and a ‘cancer’ arm, analysing both tumour and germline genomes for people affected by cancer.	100000 genomes site:[relevant URL]
Our Future Health	Our Future Health is a ‘*prospective cohort study in the UK, [that] has a mission to help people live longer, healthier lives through the discovery and testing of more effective approaches to the prevention, earlier detection and treatment of diseases. Our Future Health is a charity supported by the UK government, industry and medical charities that aims to recruit 5 million volunteers from the UK adult population to create a unique resource that will catalyse etiological and translational research for both common diseases and rare diseases*’ [13]. Full study recruitment began in 2022, aspiring to involve a diverse range of people in order to build a resource that ‘*truly reflects the UK population*’ [14]. Participants are asked to complete a baseline questionnaire, have various physical measurements, donate a blood sample (which among other tests will be used to extract DNA and conduct genotyping) and consent for linkage to health-related data [15].	‘Our Future Health’ site:[relevant URL]
Generation Study	The Generation Study is a partnership between Genomics England and the NHS that aims ‘*to screen 100,000 newborns born across England for treatable genetic disorders using whole genome sequencing as an initial test … The Generation Study aims to explore the benefits, challenges and practicalities of sequencing and analysing newborns’ genomes. As well as exploring the value of a genome-led approach to screening, it will also support healthcare research and explore attitudes and expectations around the longer term storage of genomic data to support healthcare*’ [16]. Recruitment started in October 2024 and is ongoing.	((newborn OR baby) AND (genome OR genetic OR dna OR gene)) OR ‘generation study’ OR ‘genomes programme’ OR ‘genome programme’ site:[relevant URL]
NHS genomic medicine service	Launched in 2018, aiming to build on infrastructure developed through the 100,000 Genomes Project to ensure equitable access to genomic and genetic testing across England [17, 18]. This established seven NHS Genomic Medicine Service Alliances alongside seven laboratory hubs, delivering testing as per a national genomic test directory [19].	NHS genomic medicine service site:[relevant URL]
Deciphering Developmental Disorders study	DDD is a ‘*large-scale sequencing study involving more than 13,500 families with probands with severe, probably monogenic, difficult-to-diagnose developmental disorders from 24 regional genetics services in the United Kingdom and Ireland*’ [20]. Recruitment ran from 2011 to 2015, with analysis ongoing until 2030.	DDD deciphering developmental disorder site:[relevant URL]

**Fig. 1:**
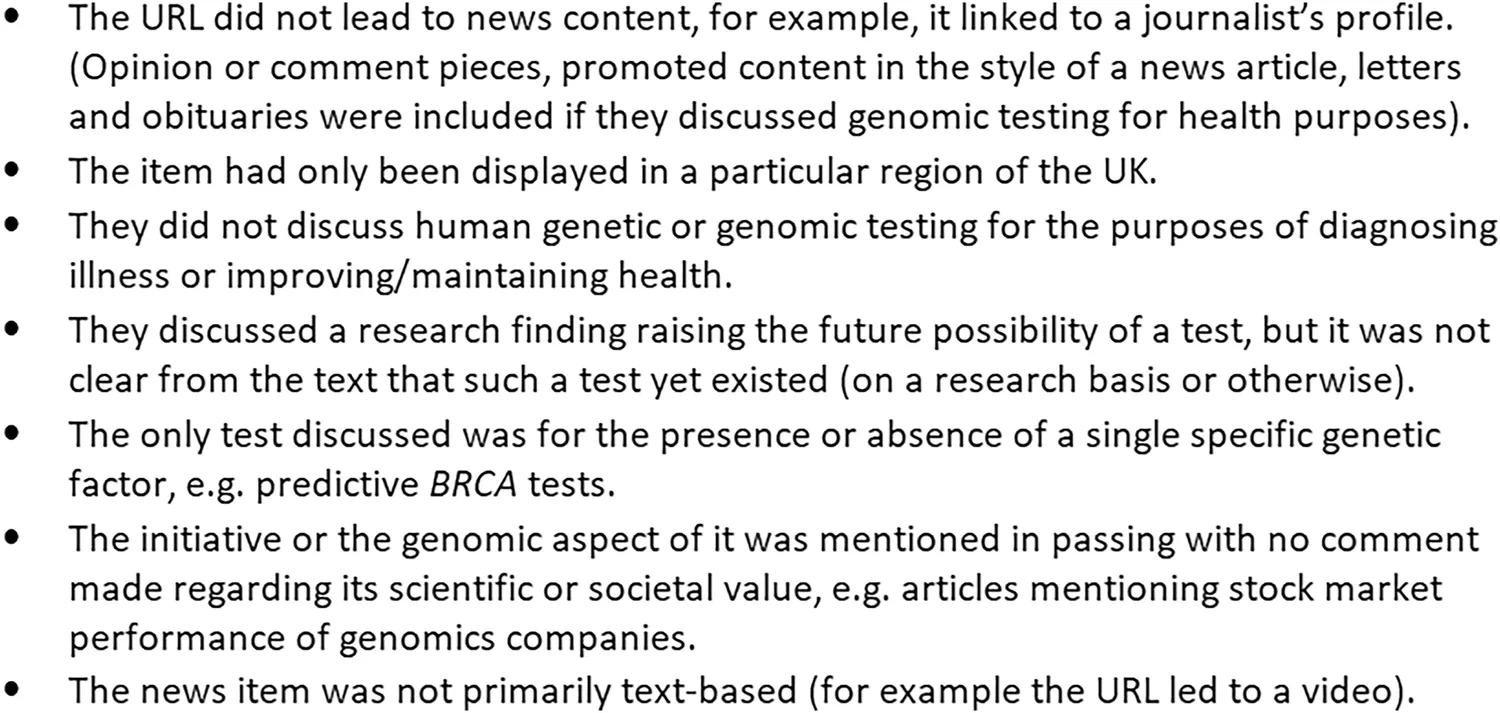
Exclusion criteria.

We ran searches with no date restrictions, allowing Google to omit results very similar to those already displayed, throughout November 2024, re-running all searches on 4/12/24 to collect items published in the intervening days. For each search, we collated results and visited each URL, checking the title of news items and, where relevant, reading their full text to assess whether they should be included in further analysis. Articles in the Times newspaper were behind a paywall, so where it was not immediately clear from a headline that an item was irrelevant, we searched for the headline in the Nexis database (https://www.lexisnexis.co.uk/products/nexis.html), which provides text-only access to many newspaper articles, in order to review it and, where relevant, collect the text. Figure 1 shows the exclusion criteria.

After collating included news items from all searches, we manually removed duplicates.

### Analysing news items

We undertook a framing analysis to explore the portrayal of genomic tests and their outcomes. Entman explains that ‘*framing essentially involves selection* *and* *salience*’ [14], so framing analysis involves noting what aspects of a story the writer has chosen to highlight or make prominent. In addition, ‘*most frames are defined by what they omit as well as include’* [14], so it also involves being alert to what is *not* reported. We chose this approach because of its scope to be informative beyond a typical content analysis as to how news items might be likely to ‘land’ with readers.

We started by reading each news item and cataloguing metadata regarding year of publication, headline, word count and name and role of the author. We also collated specific instances where genomic testing of a particular person was discussed. Using the qualitative data analysis software NVivo v15 to explore the data, we then developed inductive codes relating to the portrayal of genomic tests and results. These initially focused on very broad content of news items (e.g. ‘challenges’, ‘process of testing’).

This provided a starting point from which we developed four frames, which we went back to the news items to test, refine and explicate. Within each news item, we paid attention to which experiences around genomic tests were foregrounded and which were ignored. We considered who was quoted, the order in which quotations were presented, and what happened in stories about genomic tests (e.g. what was the outcome). See Supplementary Information for analysis of images used to illustrate news items.

## Results

### Characteristics of news items included

Searches identified 1519 results^2^, of which 250 met the inclusion criteria. After removing duplicates, 186 news items remained. Table 3 gives a breakdown of results identified and news items included by site and genomic initiative.

**Table 3:** Number of results identified via Google searches as of 4/12/24.

Project	100,000 Genomes Project		Our Future Health		Newborn Genomes Programme		NHS Genomic Medicine Service		DDD study	
Site	Screened	Included	Screened	Included	Screened	Included	Screened	Included	Screened	Included
BBC News	124	14	17	3	8	4	33	9	2	2
Sky News	34	6	2	0	8	3	8	5	0	0
Sun	47	5	10	1	8	5	13	4	1	1
Guardian	290	29	49	2	9	7	52	22	2	2
Mail	298	28	37	4	8	7	76	11	0	0
Times	199	31	35	5	8	5	35	17	2	2
Mirror	72	9	12	1	8	3	12	3	0	0

**Fig. 2:**
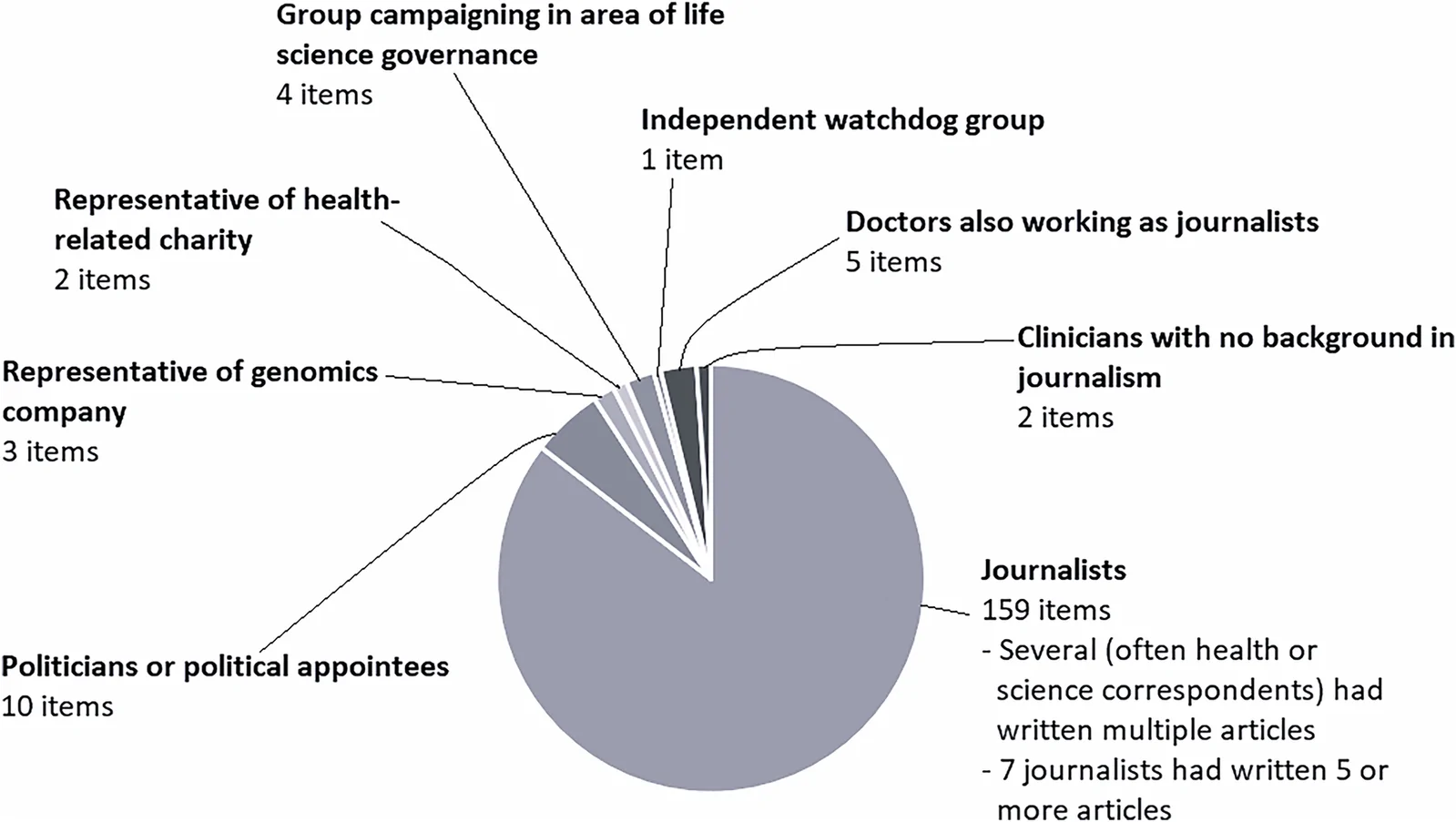
Authors of news items.

The earliest included news items were published in 2012 (announcements relating to the idea for the 100,000 Genomes Project). Figure 2 shows who authored the included news items.

News items featured in the Sun and Sky News tended to be shorter (median length 393 and 410 words respectively), with items in the Guardian/Observer and Mail being the longest (median length 749 and 873 words respectively). Table 4 shows key events reported in the news items over time: headlines give a sense of the persistent enthusiasm underlying much reporting around health-related genomic tests.

**Table 4:** Timeline of key events reported in the news items.

Date and event	Headlines around the event
August 2014 ***Announcement of 100,000 Genomes Project***	DNA project ‘to make UK world genetic research leader’ [BBC] DNA research: this is the age of the genome. And there are exciting times ahead [Observer] PM: genome project will transform cancer care [Guardian] PM Hails £300 m Project To Unlock Power Of DNA [Sky] DNA project to help target cancer care [Times]
December 2014 ***Launch of 100,000 Genomes Project***	NHS DNA scheme to fight cancer and genetic diseases [BBC] Cancer breakthrough that could spell the end of chemotherapy within 20 years: Mapping DNA could lead to faster diagnosis and new drugs [Mail] Cancer experts believe DNA study could lead to the end of chemotherapy [Mirror] DNA Study To Put UK At ‘Forefront Of Science’ [Sky] DNA testing promises healthcare revolution [Times]
January 2016 ***First diagnoses from 100,000 Genomes Project***	First children diagnosed in DNA project [BBC] First genome project diagnoses give hope to two four-year-olds [Guardian] The little girls with ‘mystery’ health conditions who finally have a diagnosis: Landmark £300 m project reads a person’s entire genetic code to detect disease [Mail] Girls’ mystery illness diagnosed through ground-breaking DNA sequencing [Times]
July 2017 ***Publication of the Generation Genome report***	Chief medical officer calls for gene testing revolution [BBC] Give patients with cancer and other serious illnesses a genetic test to create tailored treatments, says the chief medical officer [Mail] Keith Grimaldi: DNA testing for all would be a vital step in making our lives healthier and could revolutionise the NHS [Sun] (NB Keith Grimaldi is Chief Scientific Officer at DNAFit) Call for revolutionary DNA cancer care on NHS [Sky] Give every cancer patient a gene test, says health chief [Times] Genomic medicine is changing lives and Britain leads the field [Times]
July to September 2018 ***Announcement of Genomic Medicine Service***	Routine DNA tests will put NHS at the ‘forefront of medicine’ [Guardian, 3/7/18] ‘Forefront of medicine’ Patients to get routine DNA tests on NHS to tailor treatment to genes in world first [Sun, 4/7/18] NHS will become the first health service to use routine genetic testing in a move that will ‘transform patients’ lives’ [Mail, 20/9/18] DNA holy grail: NHS is to become the first health service to use routine genetic testing to make early diagnoses [Sun, 20/9/18]
January to July 2019 ***Announcements around the scheme for ‘genomic volunteers’***	NHS to offer paid-for DNA tests if patients share data [BBC, 26/1/19] NHS to sell DNA tests to healthy people in push to find new treatments [Guardian, 26/1/19] NHS to offer paid-for genetic blueprints that predict health risks [Sky, 26/1/19] Gene test for sale on NHS; Healthy patients to learn risk of killer disease [Times, 26/1/19] - Controversial plan to allow healthy patients to pay to have their DNA analysed to predict their risk of cancer, dementia and Alzheimer’s ‘breaches a core principle of the NHS’ [Mail, 29/1/19] The NHS should run a mile from the genome sequencing goldrush: David King [Guardian, 7/2/19] (NB David King is director of Human Genetics Alert) - NHS abandons plan to let healthy people pay for DNA sequencing: Amid concerns over two-tier health system, new scheme will read volunteers’ DNA for free [Guardian, 25/7/19] Millions of NHS patients will receive a FREE personalised health report based on their DNA to help them live for longer, the Government announces [Mail, 25/7/19]
November 2019 ***Plan for newborn genome screening pilot announced***	Babies’ DNA will be checked at birth in major NHS trial to ‘save thousands of lives’ by spotting genetic diseases such as epilepsy and cystic fibrosis [Mail] Brit DNA breakthrough will save thousands of kids from killer diseases [Mirror] Cot a miracle! Babies to have DNA checked at birth to identify any inherited diseases and risk of illness in later life [Sun]
June 2019 to January 2020 ***Announcements relating to exome sequencing for unwell babies***	Genome sequencing ‘revolution’ in diagnosis of sick children [BBC, 10/6/19] Seriously-ill children with mysterious conditions in England can have their entire DNA mapped as of next year in a move hoped to revolutionise the diagnosis of rare disorders [Mail, 11/6/19] Gene test hope for critically ill babies as DNA testing on the NHS could speed up diagnosis and lead to quicker treatment [Mail, 3/1/20] World first: DNA testing for critically ill babies rolled out on NHS to speed up diagnosis and treatment [Sun, 3/1/20]
April 2022 ***Tumour genome analysis finds new mutational signatures***	‘Like fingerprints at a crime scene’: study finds new clues about causes of cancer [Guardian] Cancer breakthrough as scientists find a ‘treasure trove’ of 58 new genetic clues that point to causes of the disease and raise hopes about better treatments to save more lives [Mail] ‘Treasure trove’ of DNA from tumours provides British researchers with hope for new cancer cures and clues that point to causes of the disease [Mail] NHS cancer patients will be told what caused disease thanks to ‘world-leading’ research [Mirror] Scientists find cancer ‘fingerprints’ that bring hope of new treatments [Times]
October 2022 ***Launch of rapid whole genome sequencing service***	Life-saving gene test for seriously ill children in England [BBC] New NHS genetic testing service ‘could save thousands of children’ in England [Guardian] DNA revolution ‘to save thousands of young lives’: NHS will offer blood test which can spot 6000 deadly genetic diseases within days, in world-first inspired by David Cameron’s son Ivan [Mail] ‘World-first’ NHS England rapid genetic tests ‘could save thousands of children’ [Sky]
October 2022 ***Invitations start for Our Future Health***	Millions invited to take part in UK scheme to diagnose diseases earlier [Guardian] Our health heroes! Three million Britons will join global medical study to help develop new ways to detect illnesses sooner [Mail] The Times view on Our Future Health programme: speak your weight [Times]
December 2022 ***Announcement of Newborn Genomes Programme***	Genome sequencing trial to test benefits of identifying genetic diseases at birth [Guardian] Babies will get blood tests to see if they have rare childhood cancers under major new screening drive being trialed on NHS [Mail] NHS to launch DNA test which predicts the risk of inherited diseases in newborn babies [Mirror] Cot a miracle! Newborns set to go through groundbreaking DNA tests that spot rare diseases [Sun] 100,000 newborn babies to have their DNA decoded in hunt for rare diseases [Sky] Trial to halt rare diseases in newborns [Times] Scottish newborns to miss out on new cancer screening trial [Times]
October 2024 ***First newborns recruited to Generation Study***	First newborns join screening for 200 rare diseases [BBC] NHS England to screen 100,000 babies for more than 200 genetic conditions [Guardian] DNA testing of 100,000 newborn babies begins as part of major study into 200 rare conditions [Mail] Babies set to receive a pioneering genetic test on the NHS - but many newborns will still miss out [Mirror] DNA tests: NHS hospitals screening newborn babies for rare genetic illnesses in world-first DNA study [Sun] 100,000 babies to be screened for rare genetic conditions [Times]

### Common frames by which genomic results are presented in UK news stories

#### Genomic results are the fruit of amazing technological progress and we are lucky to live at a time when we can benefit from them

Many news items contextualised contemporary genome sequencing against initiatives such as the Human Genome Project, reminding readers of the time, effort and cost it took to sequence ‘one’ genome and inviting them to share in the excitement that now, a person’s genome can be sequenced for a few hundred pounds in less than a day. Genomic results were implicitly presented as the culmination of decades of painstaking work.

A BBC News story featuring a quotation describing the 100,000 Genomes Project as ‘*transformational in what it means to society and humanity*’ described how ‘*When the first human genome was fully sequenced in 2003, it had taken 13 years and cost £2bn. Today, a genome’s worth of DNA can be sequenced in only 30 minutes, at a cost of £600*’^3^. A Mirror story started similarly:

*Reading the first-ever complete genetic blueprint for a human took 13 years of painstaking research. It was a scientific breakthrough on a par with splitting the atom and putting a man on the Moon. Today, nearly 15 years on, cutting edge technology means sequencing the three billion letters of DNA that make up a human genome takes just three days*^4^

Articles discussing rapid genomic testing of unwell children frequently contrasted a past where families waited years for answers that often did not come, with the hope that genomic testing would offer a better future. For example, a former UK prime minister wrote about the inspiration he was given by his son, who died from a rare condition. He described how the norm had been a ‘*gruelling process*’ of slow, sequential genetic testing with clinicians ‘*left to rely on intuition and antiquated tests to determine which of the 7000 rare diseases may be affecting the child*’. Writing in 2018, he described:

*Yet we are on the brink of a huge breakthrough. We have access to the comprehensive technology of clinical whole genome sequencing… Rather than testing one disease at a time, this process simultaneously can test for all rare diseases, 80 per cent of which are genetically based. All it takes is a blood test. It is that simple*^5^

The frame of how fortunate we are to now have access to previously unimaginable technology was particularly prominent in articles around newborn screening. Here, screening/prediction from the genome of a ‘healthy’ newborn was often treated as synonymous with diagnostic testing in an unwell baby. For example, a Sun article discussing newborn genome screening described: ‘*Genomic screening works by scanning DNA from a blood sample. It compares sections of genetic code to a database of illnesses to see if the patient has any. The software can check for more than 6000 conditions in days*’^6^.

News items often invited readers to conceptualise the alternative to newborn genome screening as a diagnostic odyssey of many years. For example, a BBC article discussing plans for newborn genome screening asserted: ‘*It should spare hundreds of families in England months, or years, of anguish waiting to find out why their children are ill*’^7^. As such, they created a false equivalence, setting the ‘future’ of newborn genome screening against the past of long diagnostic odysseys and glossing over the issue that the sorts of diagnoses flagged by newborn genome screening should be quickly identified by diagnostic genomic testing of a symptomatic child [15].

#### Genomic results are produced by ‘reading’ the entire genetic code

Many articles referred to genome sequencing as ‘*reading*’ the genetic code, potentially inviting an expectation that sequencing might involve understanding, not just cataloguing, of genomic variation. Several Mail articles featured an explainer that ‘*Whole genome sequencing allows researchers to read all the little bits of code that make us who we are*’^8^. A Mirror article described the 100,000 Genomes Project as ‘*aim[ing] to read the genetic blueprint, or genome, of around 75,000 volunteers*’^9^.

Some articles implied that a genome could almost tell you the answers itself. For example, a BBC News article reporting on the completion of the 100,000 Genomes Project featured the chair of Genomics England envisaging: ‘*Your health record will eventually have a genomic backbone to it; that will enable what is happening to you to be compared with what your genome is telling your doctor and therefore a more accurate diagnosis or treatment will be available*’^10^.

It was rarely made clear that obtaining a comprehensive sequence dataset and performing a comprehensive analysis, were not the same. The frequent implication was that if a genome had been sequenced it had also been ‘checked’. For example, the Times described newborn genome screening as ‘*a simple blood test [that] will look at the entire genome of healthy newborn babies in search of genetic faults in their DNA*’^11^.

When discussing genomic analysis, news items generally presented this as an enormous undertaking, but this was usually framed to maximise the marvel of the technical achievement, rather than to emphasise the corollary that people may not get clear answers from genomic tests. For example, a Times article started by telling the story of a child where genomic testing had made a treatable diagnosis and explained ‘*His DNA was extracted from a spoonful of blood and his genome– his entire genetic blueprint– was read. It is a mind-bogglingly huge task– just one person’s genetic sequence consists of enough data to almost fill an average laptop. But thanks to developments in AI and data reading, it can now be done relatively quickly and rolled out on a large scale. The results can be life-changing*’^12^.

The simplicity of sampling was frequently highlighted, with multiple articles across several news sources describing genomic tests as ‘*a simple blood test*’. The role of genomic databases was sometimes described, though often with a finality that implied that if something was logged in a database as causing disease, then this was sufficient to make a diagnosis.

Some longer articles made clear that technology plus genomes might not always equal useful results. For example, one featured stories from several families living with rare conditions undiagnosed despite extensive testing^13^. Reporting around direct-to-consumer tests was a key area where limitations were frequently raised. Several articles were written in early 2019, when the idea was floated of ‘*genomic volunteering, offering people the chance to pay to have their DNA mapped in return for voluntarily donating their anonymised data to help researchers develop treatments that will benefit everyone in the future*’^14^. These tended to discuss that making predictions from the genetic code could be difficult. However, when the plan changed from charging participants for predictions (as ‘genomic volunteers’), to providing them for free (in the context of Our Future Health), focus on this issue largely disappeared.

#### Genomic result as a panacea

The opportunity of genomic testing was often connected to a healthier and more fulfilling life. A former UK Chief Medical Officer wrote in the Times how:

*The diagnostic odyssey of being sent from medical expert to medical expert is something we all fear if we are sick. It is stressful, frustrating and most of all worrying… But imagine a world where that does not happen. One where with one blood or saliva sample a doctor could diagnose you and make sure you get the targeted treatment you need*^15^

This expectation of personal benefit from genomic testing was echoed by a former UK health secretary: ‘*Whole genome sequencing is life-changing because it is a diagnostic test of absolute certainty that can help diagnose rare diseases in weeks, not years and can help treat cancer*’^16^. Several articles connected the chance of genomic testing with increased longevity– a Mail article reporting on early plans for Our Future Health described how ‘*Millions of NHS patients will get a free personalised health report based on their DNA to help them live longer, the Government has announced*’^17^.

From the 85 instances across the news items where diagnostic genomic testing of a specific symptomatic person was discussed, 75 made a genomic diagnosis: a diagnostic rate of 88% (whilst rates quoted in clinics might be ~40% [16] and the diagnostic rate in the 100,000 Genomes Project pilot was ~25% [17]). The small ‘undiagnosed’ proportion included several people interviewed right at the outset of testing, so a reader might be left with the hope/expectation of results still to come and 4 instances where it was clear that testing had not achieved diagnosis, despite some time having passed, were all in the same article^18^. Where diagnoses had been made, many had substantially changed the person’s medical care—but stories where genomic testing had been particularly impactful were often repeated across multiple news items. Seven specific stories featured in three or more news items. All had a genomic diagnosis and for four, this had made a major difference to day-to-day medical care for themselves or their surviving family.

Genomic capabilities were often framed as essential to the survival of the NHS. A Times article reported on Our Future Health with the headline ‘*Inside the medical trial that could save us— and the NHS*’^19^. Various articles presented genomic testing as a ‘no brainer’ from a taxpayer’s perspective. For example, a Guardian article on the 100,000 Genomes Project discussed:

*As well as improving patients’ lives, WGS can also free up NHS services, saving hundreds of thousands of pounds in some cases. For instance, a 10-year-old girl who endured multiple hospital visits and intensive care admissions during her seven-year diagnostic odyssey is estimated to have cost the NHS £356,571. Once genome sequencing identified her underlying problem, the bone marrow transplant that cured her cost £70,000*^20^

The exception to this framing was articles discussing direct-to-consumer tests: their results were often represented as burdening the NHS. Similar criticisms arose relating to the 2019 ‘genomic volunteering’ scheme– when this was reborn as Our Future Health, concerns around the potential to increase strain on the NHS largely disappeared from the reporting.

#### Virtuous people would want to share their genomic data

Many stories connected participation in genomic testing with improving health care for everyone. For example, reporting on the 2019 ‘genomic volunteering’ scheme, various news outlets featured a quotation from the then UK Health Secretary that: ‘*Every genome sequenced moves us a step closer to unlocking life-saving treatments*’^21^. Contributing to a genomic database was framed as allowing ‘*groundbreaking discoveries about how diseases work, who could be susceptible to them, how we can treat them and what treatments might work*’^22^. Many articles framed a decision to share genomic data as virtuous and prosocial: one article described Our Future Health participants as ‘*Our health heroes!*’^23^.

As regards newborn genome screening, in most articles covering this there was little focus on the complexities of parents making decisions around contributing the genomic data of seemingly healthy babies. However, a few articles gave a more critical perspective. For example, a Mirror opinion piece discussed:

*A person’s genome is a huge amount of personal data and acquiring this from everyone before they’re old enough to give permission is morally questionable… One of my major concerns is the privacy and freedom of an adult being ransomed when they’re only a baby. Would that adult agree to having their genome sequenced?… I’m highly sceptical about genome sequencing of babies who have no say in the matter*.^24^

Regarding genomic data sharing in general, the Guardian/Observer ran four articles written by or heavily featuring a bioethicist with an interest in privacy and the Mail ran one, which were highly cautionary. For example, the Mail article described: *‘[bioethicist] says genomic data is ‘the oil of the digital era’ and there is nothing stopping it from be [sic] captured, bought and sold in the future’*^25^. Concerns often centred around who might have access to data, what inferences they might draw and whether these might be used against database participants. However, for most articles, if privacy/security concerns were mentioned, this was in a brief comment towards the end of the article, often followed by an assertion that in the initiative under discussion, this risk was being carefully managed.

### Absent voices

No news items made mention of the environmental impact of genomic data collection and storage [18]. The variable performance of genomic tests depending on genetic ancestry [19] was given little attention, though a few items briefly mentioned it deep within or towards the end of the article. The ‘need’ for genomes to provide genetic screening was very rarely questioned. For example, hardly any articles relating to the Generation Study/UK Newborn Genomes Programme mentioned that the newborn screening aspect of the study will only involve a tiny proportion of the genome [20]. Few news items featured people with rare conditions who had had ‘nothing found’ from genomic tests and only two included stories where the outcome of a diagnostic genomic test had been confusing or had not matched the medical issues the person was experiencing.

## Discussion

UK news items discussing health-related genomic testing tended to present this as the pinnacle of our technological prowess. NHS or university-led genomic initiatives were typically presented as almost heroic efforts that stand to give us all a better and healthier future. Many news items subscribed to a narrative that the more genomic testing we do, the better results will get, focusing on the benefits of amassing large datasets, though a few reflected that predicting future illness in a healthy person is different from explaining disease in a person who is obviously sick. Some articles explored privacy concerns relating to genomic database participation in some depth. But for the most part, people contributing to genomic databases were framed as ‘health heroes’, sharing their health information in order to reach a place where, as a society, we can all realize the benefits of genomics. The prevalent idea was of powerful and transformative genomic results. This outlook seemed shared across news sources, regardless of political leaning.

News items therefore celebrate the best of genomic results, but in doing so, they potentially imply that transformative outcomes from genomic testing come as standard, rather than being an outcome realized for relatively few [21]. This likely contributes to a climate where people have high expectations of benefit from genomic tests, increasing the intensity of disappointment arising when these are not met and potentially adding to the isolation experienced by many families affected by undiagnosed conditions [22]. The frequent tendency of news items to frame predictions from the genome as being almost synonymous with diagnoses may also impede meaningful societal discussions around what role genomics should play in population screening.

This coverage does not come from nowhere. Caulfield and Condit explored the phenomenon of science hype [23]. They traced how pressure to publish and increasing commercialisation pressure incentivises presentation of one’s research as cutting-edge and likely to imminently impact on clinical practice. Similarly, institutional press releases ‘*stimulate and shape news coverage*’ but often present research in a deterministic and simplified way. In addition, news coverage is partly determined by what readers would like to read, though may in turn influence this ‘*so that the press and the predisposed public may mutually contribute to a spiral through time*’ [23].

The highly promotional portrayal of clinical genomics that results from such processes has scope to influence people’s attitudes, albeit via more subtle ways than simply determining them [11, 24]. Sometimes such coverage will inflate expectations; in some cases, it may increase scepticism [25]. Social media are likely to play a more prominent role in the future [12], but past and present news articles provide the backdrop against which future discourses around genomic testing and its outcomes will operate.

We argue that awareness of common framings of clinical genomics in news items is important for those offering genomic tests. News coverage will not be the sole arbiter of people’s attitudes, but it may nevertheless set the scene such that many people might start consent conversations with expectations of clarity and benefit and with their decision to test, for all intents and purposes, already made. If we are alert to this, consent conversations offer a really useful opportunity to help set expectations. If not, we risk any discussion of limitations and potential downsides being purely performative- just a step to go through in order to get the transformative, potentially life-saving test of ‘*absolute certainty*’^26^ showcased by news items.

## Supplementary information

Images chosen to illustrate news items

## Data Availability

All news items discussed in this article are publicly available online (some behind a paywall). URLs, headlines and year of publication for the included news items are available on request.

## Footnotes

1 We opted to use Google for our searches rather than the Nexis database (which provides text-only access to many newspaper articles) partly because BBC News items are not catalogued on Nexis and we wanted to include these in view of their wide reach, and partly because we were keen where possible to examine the illustrative images for each article (discussed in Supplementary Information).

2 Not all of the results identified via Google search were news items. For example, some results were for a collection of links with a relevant article (captured elsewhere in the search) included, such as the sitemap for the day an article about genomic testing was published, or a biography page for a journalist including a link to an article they had written about genomic testing.

3 BBC News, *Faster diagnosis from ‘transformational’ gene project’*, 5/12/18.

4 Mirror, Britain leading the way in the fight against rare diseases with cutting edge gene machine, 30/9/17.

5 Times, *David Cameron: what I learnt from our son’s rare disease*, 28/2/18.

6 Sun, *Cot a miracle! Newborns set to go through groundbreaking DNA tests that spot rare diseases*, 12/12/22.

7 BBC News, *Newborns to get rapid genetic disease diagnosis*, 13/12/22.

8 Mail*, Babies’ DNA will be checked at birth in major NHS trial to ‘save thousands of lives’ by spotting genetic diseases such as epilepsy and cystic fibrosis*, 6/11/19; and others.

9 Mirror, *Cancer experts believe DNA study could lead to end of chemotherapy*, 23/12/14.

10 BBC News, *Faster diagnosis from ‘transformational’ gene* project’, 5/12/18.

11 Times, *Scottish newborns to miss out on new cancer screening trial*, 15/12/22.

12 Times, *Code read: The science of genomics is just a few decades old, but recent breakthroughs have meant that many children with rare diseases can now live normal lives*, promoted content with Genomics England.

13 Guardian, *‘Nobody knows what’s wrong with me’ – life with an undiagnosed condition*, 6/12/22.

14 Sky News, *NHS to offer paid-for genetic blueprints that predict health risks*, 26/1/19.

15 Times, Genomic medicine is changing lives and Britain leads the field, 4/7/17.

16 Times, *My life may have been saved this week*, 20/3/19.

17 Mail, *Millions of NHS patients will receive a free personalised health report based on their DNA to help them live for longer*, 25/7/19.

18 Guardian, *‘Nobody knows what’s wrong with me’ – life with an undiagnosed condition*, 6/12/22.

19 Times, *Inside the medical trial that could save us — and the NHS*, 28/1/23.

20 Guardian, Whole genome sequencing could save NHS millions of pounds, study suggests, 10/11/21.

21 BBC News, *NHS to offer paid-for DNA tests if patients share data*; Guardian, *NHS to sell DNA tests to healthy people in push to find new treatments*; Times, *Gene test for sale on NHS: healthy patients to learn risk of killer diseases*; Sky News, *NHS to offer paid-for genetic blueprints that predict health risks*; all 26/1/19.

22 Observer, *What I learned from home DNA testing*, 23/7/17 (the comment was made in reference to the 100,000 Genomes Project, not ‘home DNA testing’).

23 Mail, Our health heroes! Three million Britons will join global medical study to help develop new ways to detect illnesses sooner, 24/10/22.

24 Mirror, Harvesting baby DNA to uncover potential diseases is an invasion of our privacy, 4/4/22.

25 Mail, Google could soon get access to hundreds of thousands of patients’ genetic data: An expert explains why we should be concerned, 5/7/17.

26 Times, *My life may have been saved this week*, 20/3/19.
