# Supplementary material for: Transforming our future by unlocking the power of DNA: the representation of health-related genomic testing in UK news items
Source: Eur J Hum Genet. 2026 May 21;34(10):1554–63. doi: 10.1038/s41431-026-02140-8 (PMC13634045; doi:10.1038/s41431-026-02140-8)
Supplement: Supplementary file 1 [file 41431_2026_2140_MOESM1_ESM.docx]

**Supplementary Information to *‘Transforming our future by unlocking the power of DNA: the representation of health-related genomic testing in UK news items’***

***Images chosen to illustrate news items***

In earlier work we examined the representation of genetic testing in stock image banks(1). This was partly motivated by noticing that stock images were fairly often used to illustrate news stories about genetic/genomic testing. For many people browsing the news sources where the items we discuss were featured, the headline and an illustrative image may have been all they saw. As part of our analysis we therefore wanted to explore which images had been used to illustrate included news items. Due to word count constraints we present this here.

**Methods**

Image collection

Part of our rationale for using Google rather than the Nexis database to run searches was that Nexis provides text only versions of articles, whereas via Google, we could easily access news items as presented by the relevant news outlets, including any images used to illustrate them. The exception was Times news items which were behind a paywall, and the 47 Times news items included in the analysis we present in the main article were therefore excluded from this analysis.

Image analysis

Our analytic approach was informed by our earlier work exploring the representation of genetic testing in stock image banks(1). We took a two-step approach to analysing images, first focusing on content. For example, we considered what was depicted (e.g. double helix; scientific equipment; ‘real’ person experiencing genomic testing) and details relating to this (e.g. if a person was pictured, what was their facial expression? What was the setting (home, hospital, etc)?). We then aimed to consider what messages such content might communicate to a viewer, in combination with the article text.

Bearing in mind our overarching framing analysis approach, we also considered which images were given primacy (e.g. which featured at the top of articles, and so likely featured as a ‘thumbnail’ image visible to people who did not go onto read the associated item at the time it was prominent on the news source (i.e. around the time of publication)).

**Results**

For 139 news items, we were able to view the content such that we could see accompanying images: all of these had at least one illustrative image. Supplementary Table 1 shows common content in illustrative images.

*Supplementary Table 1: Common content in images illustrating included news items*

| **Content** | **Number of news items (percentage)** | **Notes on presentation** |
| --- | --- | --- |
| Double helix | 38 (27%) | Usually blue, often glowing. Where featured this was often the first image of the article (so likely to have been the thumbnail). |
| Scientific equipment (e.g. pipette, microscope, sample tubes) | 27 (19%) | Where equipment was featured it was often the focal point of the image. |
| Slab gel electrophoresis-type pattern | 12 (9%) | Bands were usually starkly delineated and sometimes glowing. |
| ACGT letters | 2 (1%) | In one image, white ACGT letters filled the background and in the foreground was a gloved hand holding a magnifying glass, in the lens of which were coloured ACGT letters surrounding the word ‘Cancer’^[1]^ |
| Act of sampling (blood tests, cheek swabs, heel pricks) | 15 (11%) | These tended to be cropped such that little could be seen of the person having the test, e.g. just the antecubital fossa with a needle in it; the foot of a newborn baby after a heel prick. |
| Politician | 17 (12%) | Usually UK health secretaries or prime ministers. |
| ‘Real people’ experiencing genomic testing | 42 (30%) | See section below. |

**‘Real people’ experiencing genomic tests**

42 news items (30%) featured images involving people or families who had been affected by genomic testing, for example children who had had genomic testing aiming to diagnose a rare condition, or adults who had signed up to participate in Our Future Health (in this category we are not counting stock images, for example stock ‘newborn baby’ photos which illustrated several news items on newborn genome screening).

Images of ‘real people’ were most common for BBC News articles, where 65% featured at least one photo of a ‘real’ person who had been in some way affected by genomic testing, and least common for Guardian/Observer articles where this applied to only 9%. The Mail and Mirror featured photos of ‘real’ people in 36% and 38% of news items respectively, but where they did so they tended to show multiple photos. For example, one Mail news item featured 11 photographs of ‘real’ families who would likely have had better outcomes if genetic diagnoses had been made earlier^[2]^ – the other image on the page showed a newborn baby with an annotated glowing double helix hovering over their cot (the thumbnail image for a linked article about newborn genome screening^[3]^).

Where articles showed only one or a few pictures of a ‘real’ person or family affected by genomic tests, these tended to look either like smiley family photos or would show the people involved with a relatively neutral but thoughtful expression. Sometimes these photos appeared to be taken at home, for example a family might be sitting together on the sofa and smiling at the camera, and sometimes they looked like holiday snaps, for example a child with their parents at the beach.

Where articles featured multiple photos, these tended to be a selection that on the one hand symbolised ‘everyday family life’, for example parents playing with their child in their living room, siblings smiling together in a garden, together with a few separate images that emphasised severe illness and vulnerability, for example a small child sleeping in a big hospital bed, surrounded by drips and tubes. Looking at them as a set, they gave the impression of a family ricocheting between terrible stress and happy family life – with the implication being that genomic results were key in shifting them from the overwhelming illness made evident in certain of the photos, to the smiling family whose photo was at the top of the news item in question.

**Discussion**

Images can be consequential(2). As regards genomic testing, illustrative images may often be chosen as an afterthought, but they may yet convey ideas around what to expect from genomic tests. This is perhaps especially the case when partnered with text which, as we discuss in our main article, tends to foreground genomic testing at its very best, and often ignores disappointing or unhelpful outcomes.

As Messaris explains when introducing his book *Visual Persuasion: The Role of Images in Advertising*, ‘*because of the implicit nature of visual argumentation and the relative lack of social accountability which images enjoy in comparison with words, pictures can be used to make advertising claims that would be unacceptable if spelled out verbally*’(3). Various of the illustrative images, particularly the frequent use of a glowing double helix as the most prominent image in news items, may promote an idea of genomic results as powerful, profound and packed with insight as a matter of course(1).

Providers of genomic tests may wish to carefully consider which illustrative images they choose to use in the course of their work.

**References**

1.	Horton R, Boyle L, Weller S, Lucassen A. Glowing gels and pipettes aplenty: how do commercial stock image banks portray genetic tests? Eur J Hum Genet. 2024 Apr;32(4):456–60.

2.	Haelle T. Tips on picking good photos for vaccine stories [Internet]. Association of Health Care Journalists. 2021 [cited 2025 Aug 1]. Available from: https://healthjournalism.org/blog/2021/06/tips-on-picking-good-photos-for-vaccine-stories/

3.	Messaris P. Visual Persuasion: The Role of Images in Advertising. SAGE; 1997. 324 p.

1. BBC News, *Chief medical officer calls for gene testing revolution*, 4/7/17
2. Mail, *Our children were handed a death sentence when a simple blood test at birth could have saved their lives - by the time doctors took us seriously, it was too late for treatment*, 5/11/24
3. Mail, *DNA testing of 100,000 newborn babies begins as part of major study into 200 rare conditions*, 3/10/24
